# Relationship between psychosocial dimensions of personality and perception of anxiety, with injury history in elite athletes

**DOI:** 10.3389/fspor.2026.1721355

**Published:** 2026-02-11

**Authors:** M. Peñaranda-Moraga, José Manuel García-De Frutos, Yolanda Nadal-Nicolás, Bernardo J. Cuestas-Calero, Alejandro Martínez-Rodríguez, Rodrigo Yáñez-Sepúlveda, Guillermo Cortés-Roco, José Francisco López-Gil, A. J. Muñoz-Villena

**Affiliations:** 1 Department of Analytical Chemistry, Nutrition and Food Science, University of Alicante, Alicante Institute for Health and Biomedical Research (ISABIAL), Alicante, Spain; 2 European Institute of Exercise and Health (Spin-Off University of Alicante), Alicante, Spain; 3 Facultad de Deporte, Universidad Católica San Antonio de Murcia, Murcia, España; 4 Translational Research Center in Physiotherapy, Department of Pathology and Surgery, Faculty of Medicine, Miguel Hernandez University, Alicante, Spain; 5 Faculty Education and Social Sciences, Universidad Andres Bello, Viña del Mar, Chile; 6 Universidad Viña del Mar, Facultad de Ciencias de la Vida, Programa de Magíster en Evaluación y Planificación del Entrenamiento Deportivo, Carrera de Entrenador Deportivo, Viña del Mar, Chile; 7 School of Medicine, Universidad Espíritu Santo, Samborondón, Ecuador; 8 Vicerrectoría de Investigación y Postgrado, Universidad de Los Lagos, Osorno, Chile; 9 Department of Communication and Social Psychology, University of Alicante, Alicante, Spain

**Keywords:** character, injury, psychosocial, team sports, trait anxiety

## Abstract

**Introduction:**

High-performance sports practice involves both physical challenges and psychosocial factors that influence the performance and well-being of athletes. In this context, both genetic and cultural/social dimensions of personality and the perception of anxiety play a fundamental role in the sporting experience, especially when related to the history of sports injuries.

**Methods:**

Thus, the objective of this research was to analyze the dimensions of personality and anxiety in a sample of team athletes, as well as to examine the relationships between these psychosocial variables and their predictive capacity in relation to the severity of sports injuries. A battery of questionnaires was administered to a sample of 63 elite athletes (55.6% men, 44.4% women), aged between 18 and 36 years (M = 22.62; SD = 4.26) and belonging to different team sports.

**Results:**

The results indicated that there are significant differences in the psychological dimensions according to the history of injuries. Somatic and cognitive anxiety were significantly associated with the presence and severity of injuries. In addition, temperamental and character dimensions were identified as predictors of injuries.

**Discussion:**

These findings suggest that psychosocial variables play a key role in the occurrence and severity of injuries, which may be fundamental for both future research and for professionals applied to high-performance sport..

## 1 Introduction

In recent decades, the study of the relationship between psychological factors in athletes and vulnerability to injury has generated a solid body of knowledge, supported by reviews (1–5) and numerous empirical studies (6–14). These lines of study have made it possible to identify how the psychosocial characteristics of athletes significantly influence various aspects related to sports injuries, including the likelihood of suffering them (1, 5, 14, 15), the rehabilitation and recovery process (16–18), and adherence to treatment (2).

Various theoretical approaches have attempted to establish links between psychological variables and susceptibility to injury. These include the recursive model of injury a etiology by Meeuwisse et al. (19), the risk profile approach by Wiese-Bjornstal (15), and one of the most influential, the Stress-Injury model by Andersen and Williams (20). A more updated version of the latter proposal (21) or the biopsychosocial model proposed by Olmedilla-Zafra and García-Mas (22) have provided a useful conceptual framework for explaining the role of individual differences, such as personality, in cognitive and physiological responses, and their influence on the physical vulnerability of athletes to injury.

The study of personality in the sporting context has been one of the most researched topics and has often generated considerable controversy due to the diversity of theoretical models and results obtained. This complexity is also reflected in the approaches taken to identify the influence of personality on sports injuries (23, 24). In this area, studies have analyzed the indicators proposed by the stress-injury model and their relationship with individual differences such as resilient personality (25), perfectionism (11, 26, 27), competitiveness (10), tension and daring (28) and neuroticism (29).

In most cases, personality variables have shown, to varying degrees, a consistent relationship with injury vulnerability, highlighting the need to address them using more current and advanced models. Recent empirical evidence suggests that this relationship is neither direct nor linear, but rather mediated by a series of interrelated cognitive, emotional, and behavioral mechanisms. Specifically, athletes with higher levels of neuroticism or anxiety tend to perceive competitive situations as more threatening, exhibit greater difficulties in emotional regulation, and show alterations in attentional control and decision-making under pressure, which increases the likelihood of technical errors, maladaptive motor responses, and excessive physiological arousal (29, 30). Additionally, psychological inflexibility has been linked to less effective management of pain, fatigue, and adversity, fostering maladaptive perseverance patterns or avoidance behaviors that can increase exposure to situations of physical risk (29, 31). In sports characterized by high physical demands, speed, and technical complexity, these mechanisms take on particular relevance, since small psychological alterations can significantly amplify vulnerability to injury. From this perspective, contemporary approaches allow for a more complex analysis by integrating both the basic dimensions of personality and their interaction with contextual, emotional, and physiological factors.

In this regard, the psychobiological model of personality proposed by Cloninger (32), when applied to the sport context, establishes a fundamental distinction between temperament and character traits. Temperament is associated with a biological and hereditary basis and influences, from the early stages of development, how athletes perceive, process, learn, and memorise information, determining which stimuli are considered relevant. Character, in contrast, is progressively constructed through social and cultural experiences and modulates the emotional and behavioural responses derived from temperament. Cloninger (32) proposes the existence of a bidirectional interaction between these two components, whereby temperamental dispositions condition character formation, while character, in turn, regulates and adjusts the expression of temperament, generating new patterns of behaviour in the athlete. As a result of this dynamic interaction between biological factors and environmental experiences, individual differences in athletes’ personalities emerge, influencing how they cope with competitive demands, make decisions, and manage stressful situations, with potential implications for both sport performance and the likelihood of sustaining injuries.

Despite the usefulness of Cloninger's model for studying the relationship between personality and sports injuries, research applying it in this context, connecting psychosocial and physical- biological constructs, has been limited to date (33–36). However, this model is particularly relevant and valuable, as it allows for a detailed assessment of temperamental traits that may predispose an athlete to situations of greater physical risk, such as the tendency to seek novelty (33) or avoid harm (34). For example, Baba (33) found in a sample of marathon runners that Novelty Seeking was positively associated with ligament and tendon injuries, while Reward Dependence was linked to asthma. Similarly, McFie et al. (34) identified a negative relationship between Harm Avoidance and susceptibility to concussion in a sample of rugby players.

In relation to character traits, Monasterio et al. (36), using a sample of mountaineers, found an inverse relationship between Cooperation and the frequency and severity of injuries. Meanwhile, research by Con et al. (37) has confirmed that other traits such as Self-Direction and Cooperation influence an athlete's ability to manage levels of anxiety and depression. Monasterio and Cloninger (35) found in a sample of jumpers, mountaineers and the general population that low scores in Self-Transcendence were associated with risk-taking behaviours.

Anxiety has been identified as a determining psychological factor in the study of the incidence and recovery of sports injuries, and this has been widely confirmed in numerous studies (7, 29, 38–41). This research has corroborated that high levels of anxiety increase the risk of injury due to physiological and cognitive responses that affect the athlete's ability to adapt to the demands of the competitive sporting environment.

In this context, athletes face a series of physical, psychological and social stimuli that require adaptive responses in order to achieve their goals. These responses, understood as reactions to stimuli, can be physiological (heart rate, sweating, release of neurotransmitters) or psychological (emotions, beliefs, behaviour, motivation). Thus, depending on how the athlete perceives and interprets these stimuli as threatening or not, different types of responses will be activated that can influence both their physical performance and vulnerability to injury, as well as their ability to recover.

According to Anderson and Williams’ stress-injury model (1998), anxiety emerges as a central component of the stress response, directly influencing the risk of injury. This approach suggests a bidirectional relationship between individual cognitive assessment and the psychological and attentional factors of stress. Thus, when an athlete perceives their resources as inadequate to meet the demands of a situation, they consider relevant to success, a stress response characterized by high anxiety intensity is activated. This activation, in turn, triggers physiological changes (such as muscle tension) and attentional alterations (such as narrowing of the attentional focus) that affect the athlete's ability to respond, increasing vulnerability to injury.

In this vein, anxiety has been defined as a psychological response to a perceived threat, characterized by negative affectivity and high psychophysiological arousal, resulting from the perceived discrepancy between environmental demands (sporting pressure) and the athlete's self- assessment of their own abilities (42). Specifically, competitive trait anxiety represents a tendency to perceive competitive situations as threatening, constituting a relevant factor due to its individual stability and predictive capacity regarding state anxiety.

For decades, theories studying the relationship between anxiety and performance have varied, with those taking a multidimensional approach currently standing out. Multidimensional theory (43) identifies three factors (somatic anxiety, cognitive anxiety, and self- confidence) as predictors of athletic performance. Martens et al. (43) define anxiety as the combination of a somatic or physiological reaction related to the affective and physiological elements of the experience of anxiety derived from autonomic activation, and a cognitive reaction consisting of negative expectations and concerns about oneself, the situation, and possible consequences. Self-confidence refers to the degree of conviction an athlete has about their ability to achieve success in their performance.

To this approach, we add the approach of Jones and Swain (44), who introduce the concept of anxiety direction/valence. Unlike previous theories that have considered anxiety to be a negative and maladaptive trait, this new perspective proposes that anxiety depends on the individual's interpretation of it as a limiting or facilitating factor in performance. Thus, the intensity of anxiety corresponds to the level of symptoms experienced, while direction/valence refers to the athlete's interpretation of anxiety symptoms as limiting or enhancing.

This conceptual framework proposed by Jones and Swain (44) has provided a useful perspective when it includes individual differences in the anxiety process and how these personal characteristics can influence the individual's interpretation of their symptoms in relation to performance. Consequently, the valence/direction dimension of anxiety would play a decisive role in understanding why two people may experience the same intensity of anxiety at a given time and in a given situation, yet differ in their interpretation of the symptoms. In this sense, athletes perceive the intensity of their symptoms and interpret whether they will be an obstacle or a facilitator to their competitive performance, based on their perception of control and personal ability. The athlete's degree of control refers to the possibility of making changes to the context and to themselves, while ability implies the competence to face difficulties in achieving their goals.

Athletes repeatedly face sporting and competitive situations under high levels of both internal and external pressure, where their personal resources for coping and their perception of these situations are decisive. It is then that personality, as a psychological system, intervenes in the interaction-adaptation between the athlete and the environment, mediating and evolving in their relationship with other affective, cognitive and behavioural processes that affect their vulnerability to injury.

In view of these reasons, the stress-injury model of Williams and Andersen (21) provides a conceptual framework on which to base the study of these variables. According to this model, the risk of injury is related to the subjective perception of threat, the assessment of personal resources and the coping resources of each athlete. The ability to self-regulate behaviour and adapt to a demanding environment allows athletes to manage their perception of control in order to moderate their emotional response (anxiety) to stressful situations. Thus, athletes with a high capacity for self-regulation and self-direction tend to interpret anxiety as a manageable challenge rather than a threat, which reduces the likelihood of maintaining a state of high stress and decreases the risk of injury.

Various studies highlight how individual differences in personality condition athletes’ psychological responses to threatening situations or physical vulnerability, such as injury (4, 11, 28, 29, 31, 33, 34). Thus, in the interaction between temperamental traits and learning experiences that shape character, personality-related responses shape the way athletes cope with stress. These factors largely determine athletes’ ability to functionally adapt to the demands of the competitive environment, optimising or limiting their potential, depending on their interpretation of the threatening situation.

Based on the literature reviewed, a cross-sectional study with a non-randomised, correlational and predictive design was proposed. The purpose of this study was, on the one hand, to describe the dimensions of personality and anxiety in a sample of team athletes based on their injury history and, on the other hand, to analyze the relationships between psychosocial variables and their predictive capacity with regard to the severity of injuries.

The initial hypotheses proposed that athletes with a history of injuries would show lower scores in Harm Avoidance (34), Self-Transcendence (35) and Cooperation (Monasterio et al., 201); and higher scores in Novelty Seeking (33). In addition, athletes with higher levels of anxiety, both somatic and cognitive, would show greater vulnerability and incidence of injuries (7, 38–41).

## 2 Methodology

### 2.1 Participants

The sample consisted of 63 athletes belonging to professional handball clubs (ASOBAL League and Guerreras Iberdrola), football clubs (Second Federation) and rugby clubs (Honour Division) in the provinces of Alicante and Murcia. The participants’ ages ranged from 18 to 36 years (M = 22.62; SD = 4.26). In terms of gender, there were 35 males (55.6%) and 28 females (44.4%); while, in terms of sport, 11 were rugby players (17.5%), 36 were handball players (57.1%) and 16 were football players (25.4%). In terms of injury history, 10 athletes (15.9%) had not suffered any injuries, 6 athletes (9.5%) had minor injuries, 28 athletes (44.4%) moderate injuries, 15 athletes (23.8%) serious injuries and 4 athletes (6.3%) very serious injuries.

### 2.2 Instruments

To assess the different variables under study, the following assessment instruments were administered to participants and professionals:

Questionnaire on injury history. This self-report instrument was used to record the frequency, severity, and type of injury suffered by the athletes. A sports injury was defined as any physical impartiment or problem occurring during training or competition that resulted in at least one day of partial or total inability to participate in sports. To assess the incidence of injuries, athletes were asked to indicate the number of injuries that occurred during the sports season. More specifically, to assess the severity of the injury, a functional criterion established by Olmedilla et al. (22) was used to differentiate between minor injuries (at least interrupting one day of training and requiring treatment), moderate injuries (forcing the athlete to interrupt their training and competitions for at least one week and requiring treatment), serious injuries (involving one or more months of sports leave, sometimes hospitalisation, even surgery) and very serious injuries (causing a permanent decrease in the athlete's performance, requiring constant rehabilitation to prevent worsening). On the other hand, to assess the type of injury, a classification was used that included muscle injuries, fractures, tendinitis, contusions, sprains and others.

Competitive Trait Anxiety Inventory (CTAI-2D): adapted for Spanish athletes by Muñoz-Villena et al. (45). This instrument consists of 27 items that assess three factors: cognitive anxiety (e.g., item “I worry about not doing as well as I could”), somatic anxiety (e.g., “I feel nervous before the competition”), and self-confidence (e.g., “I usually feel relaxed before competing”); and two scales: intensity and valence/direction. On the one hand, intensity is assessed using four response options presented on a 4-category Likert scale, where 1 corresponds to “not at all” and 4 corresponds to “very much”. On the other hand, for valence/direction, the response options are presented on a 7-category Likert scale, ranging from −3 “very negative for athletic performance” to +3 “very positive for athletic performance”. ’s alpha reliability offered by the scales ranges from.74 for the somatic anxiety scale (intensity) to.96 for the cognitive anxiety scale, indicating an overall consistency of.82.

Temperament and Character Inventory [TCI-R-67; (46)]. Abbreviated version adapted to Spanish of the self-report consisting of 62 items (plus 5 validity items), which are answered on a 5-point Likert scale, ranging from 1 (strongly disagree) to 5 (moderately agree), according to the 7 dimensions described in Cloninger's model. The instrument measures four temperament dimensions: *Novelty Seeking*, defined as a tendency toward exploratory behaviour and impulsivity (e.g., “*I often spend money until I have nothing left or go into debt by taking on too many credits*”); *Harm Avoidance*, reflecting a predisposition to risk avoidance and fear of uncertainty (e.g., “*I often feel tense and worried in unfamiliar situations, even when others think there is no danger*”); *Reward Dependence*, referring to a tendency to respond strongly to social approval and emotional support (e.g., “*I like to talk openly with my friends about my experiences and feelings rather than keeping them to myself*”); and *Persistence*, defined as the capacity to maintain effort despite frustration or fatigue (e.g., “*I enjoy challenges more than difficult tasks*”). In addition, the inventory assesses three character dimensions: *Self-Directedness*, which reflects the ability to regulate and adapt behaviour in accordance with personal goals and values (e.g., “*I often think that my life has little meaning or purpose*”); *Cooperativeness*, referring to empathy, tolerance, and helpfulness in social interactions (e.g., “*I have little patience with people who do not accept my point of view*”); and *Self-Transcendence*, defined as the tendency to experience a sense of connection beyond the self (e.g., “*At times I have felt that I am part of something that has no limits or boundaries in space or time*”). The overall reliability index for this research was.75.

### 2.3 Procedure

To carry out this research, a convenience sample was taken from the elite handball, football and rugby clubs where the researchers carry out their professional activity. In both cases, the method of data collection and the objectives of the research were explained in detail to both the coach and the physiotherapists. Next, the athletes, all of legal age, were informed of the voluntary nature of their participation, the confidentiality and anonymity of the data, ensuring greater sincerity. The battery of questionnaires was administered collectively in the changing room where each team usually meets, with approximately half an hour spent between the researchers explaining the instructions and the time taken by the participants to answer the instruments. The researchers have ensured compliance with the Helsinki Declaration (2000) for this study, together with the consent of the athletes and the approval of the Ethics Committee of the University of Alicante (UA-2019-04-09).

### 2.4 Data analysis

Data coding and processing were performed using the R statistical package for Windows. Descriptive analyses of central tendency (mean and standard deviation) and frequencies were performed on the psychological variables and injury history, respectively. In addition, an internal reliability analysis of the measures used (Cronbach's alpha) was performed, together with consideration of the normal distribution (Kolmogorov–Smirnov) of the variables investigated and the comparison of means based on whether or not the athlete had suffered an injury (ANOVA and t-student). When significant main effects were identified in the ANOVA, *post hoc* comparisons were performed using Tukey's test to determine specific group differences. The relationship between the variables under study was explored using a correlational analysis (Pearson) and their predictive level on the severity of the injury was determined through a multinomial logistic regression analysis. All analyses were performed with a significance level of.05.

## 3 Ethics statement

The studies involving human participants were reviewed and approved by the University of Alicante Ethics Committee (UA-2019-04-09). All participants provided written informed consent in accordance with the Declaration of Helsinki (2000).

## 4 Results

### 4.1 Descriptive analysis

Table 1 shows the descriptive statistics (mean and standard deviation) and the results of the normality test for the variables under study, differentiated according to the severity of the athlete's injury. Analysis of variance (ANOVA) in personality dimensions shows significant differences in the means of the scores for Novelty Seeking (F = 3.957); *p* = .000), Persistence (F = 3.620; *p* = .011) and somatic anxiety intensity (F = 3.44; *p* = .014).

**Table 1:** Descriptive statistics of the variables under study based on injury history.

Variable	*α*	K-S	WI (*n* = 10)	MI (*n* = 6)	MO (*n* = 28)	S (*n* = 15)	MS (*n* = 4)
			M(DT)	M(DT)	M(DT)	M(DT)	M(DT)
NS	.63	.08	21.20 (2.25)	21.67 (3.88)	18.82 (3.94)	18.20 (2.88)	14.50 (1.91)
HA	.73	.06	18.80 (2.34)	16 (.89)	20.32 (6.27)	21.07 (3.36)	19.50 (1.91)
RD	.85	.05	26.80 (4.39)	22.50 (5.61)	25.64 (6.14)	25.13 (8.23)	19.25 (7.63)
P	.80	.06	26.80 (3.08)	28.53 (3.31)	31.54 (4.31)	30.73 (3.77)	33.25 (2.36)
C	.73	.05	26.60 (2.87)	29.33 (4.22)	29.54 (4.46)	29 (5.09)	31.50 (4.72)
SD	.74	.06	31 (3.19)	32.83 (1.47)	32.18 (4.20)	32.37 (2.94)	34.25 (3.5)
ST	.81	.07	16.20 (4.34)	20.50 (3.88)	17.86 (4.46)	19.53 (6.81)	14.50 (2.51)
SA-I	.80	.20	16.20 (3.29)	17.33 (3.38)	20.86 (5.20)	23.47 (6.09)	19 (3.46)
CA-I	.90	.20	23.40 (4.3)	24.17 (2.48)	26.25 (4.99)	28.07 (4.54)	25.75 (7.50)
SELF-I	.76	.20	25.80 (3.15)	29.83 (4.35)	24.32 (7.12)	21.20 (7.15)	26.25 (.50)
SA-D	.81	.20	3.20 (9.87)	9.83 (8.13)	5.50 (10.60)	5.40 (8.89)	.25 (10.30)
CA-D	.94	.20	3.40 (5.71)	4.33 (4.84)	6.21 (10.43)	5.93 (9.55)	6.25 (9.50)
SELF-D	.77	.20	10(7.33)	16.33(4.80)	7.14(10.08)	4.60(12.47)	10.75(1.89)

NS, novelty seeking; HA, harm avoidance; RD, reward dependence; P, persistence; C, cooperativeness; SD, self-directedness; ST, self-transcendence; SA-I, somatic anxiety (intensity); CA-I, cognitive anxiety (intensity); SELF-I, self-confidence (intensity); SA-D, somatic anxiety (direction/valence); CA-D, cognitive anxiety (direction/valence); SELF-D, self-confidence (direction/valence); WI, without injury; MI, mild injury; MO, moderate; S, severe; MS, more severe.

Post hoc analyses using Tukey's HSD test were conducted to identify specific group differences according to injury severity. Regarding the temperamental dimension of Novelty Seeking, athletes who had sustained very severe injuries showed significantly higher mean scores compared to both non-injured athletes (*p* = .012) and those with mild injuries (*p* = .015). This finding suggests that a greater tendency toward exploration, impulsivity, and risk-taking is associated with higher levels of injury severity. With respect to Persistence, *post hoc* comparisons revealed that non-injured athletes reported significantly higher scores than athletes who had experienced moderate injuries (*p* = .012) and very severe injuries (*p* = .048). This pattern indicates that lower levels of perseverance and sustained effort tolerance may be linked to increased injury severity. Finally, concerning anxiety dimensions, Tukey *post hoc* analyses showed that athletes who had suffered a severe injury exhibited significantly higher levels of somatic anxiety intensity compared to those who reported no injuries (*p* = .011), highlighting the role of heightened physiological arousal under stress in athletes with more severe injury outcomes.

Depending on the type of injury, differences were found in Reward Dependence, Persistence, Self-Transcendence, Somatic Anxiety and Cognitive Anxiety (direction). In Reward Dependence, differences were found between those with muscle injuries and those with sprains, with the latter scoring higher. In Persistence, there are differences between those with muscle injuries and those with sprains and fractures. In Self-Transcendence, there are differences between those with fractures and those with sprains and contusions. In terms of the dimensions of anxiety (valence/direction), somatic anxiety shows differences between those with fracture injuries and those with contusions or sprains, with the latter having higher scores. In cognitive anxiety (direction), there are significant differences between those suffering from tendinitis and muscle injuries and sprains, with higher scores in the latter.

**Figure 1:**
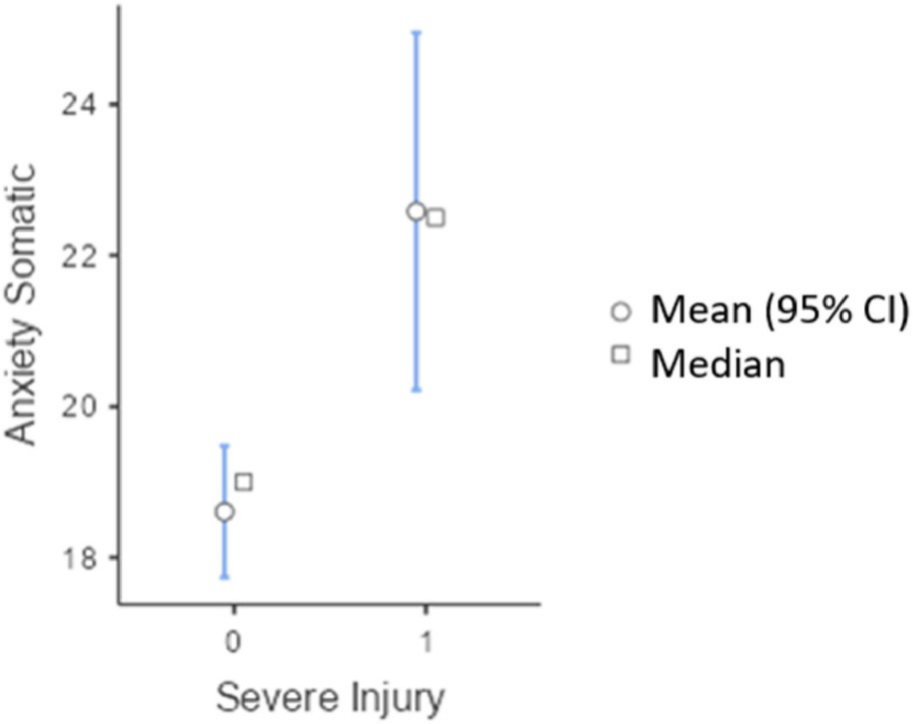
Descriptive graph of the intensity of somatic anxiety based on injury or no injury.

On the other hand, with regard to the presence or absence of injury, the analysis of means corroborated significant differences in the dimensions of Novelty Seeking, Persistence, Somatic Anxiety (intensity) and Cognitive Anxiety (intensity). Specifically, in terms of personality dimensions, athletes who have not suffered an injury during the season show higher levels of Novelty Seeking (t = 2.055; *p* = .044); on the other hand, those who have suffered an injury show higher levels of Persistence (t = −3.243; *p* = .002). In relation to anxiety, Figure 1 compares the perception of somatic anxiety based on the absence or presence of injury, showing significantly higher scores in athletes who had suffered an injury in contrast to those who had not (t = −3.66; *p* = .008), with a large effect size (d = −.83).

**Figure 2:**
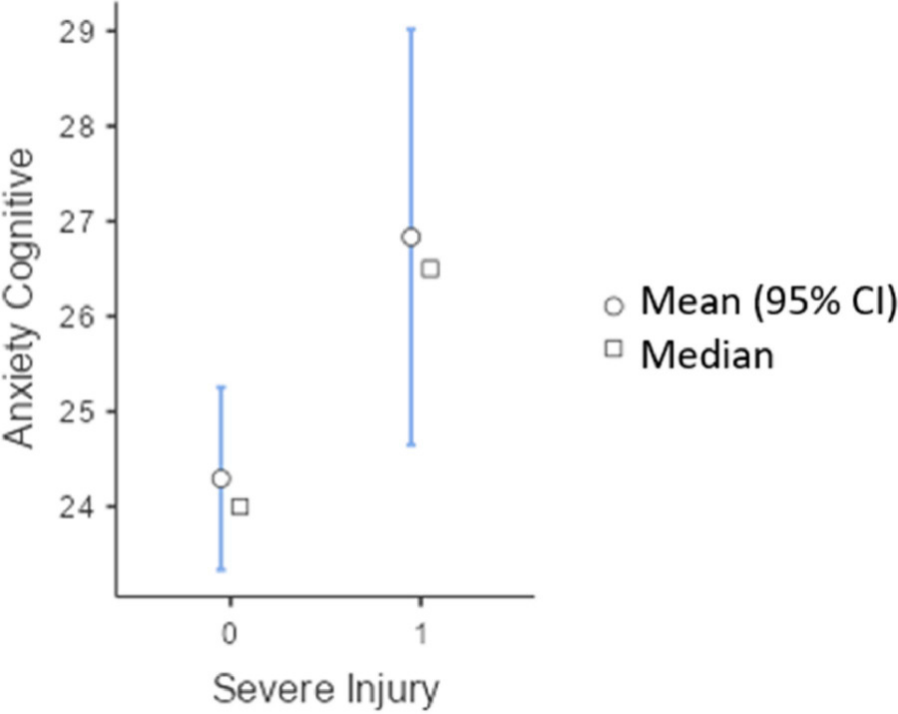
Descriptive graph of the intensity of cognitive anxiety depending on whether or not the athlete had suffered an injury.

Similarly, Figure 2 shows the differences in the intensity of cognitive anxiety, demonstrating that athletes who had suffered an injury (t = −2.22; *p* = .022) had more concerns and doubts about their performance compared to those who had not suffered an injury, with a moderate effect size (d = −.50).

### 4.2 Relational analysis

**Figure 3:**
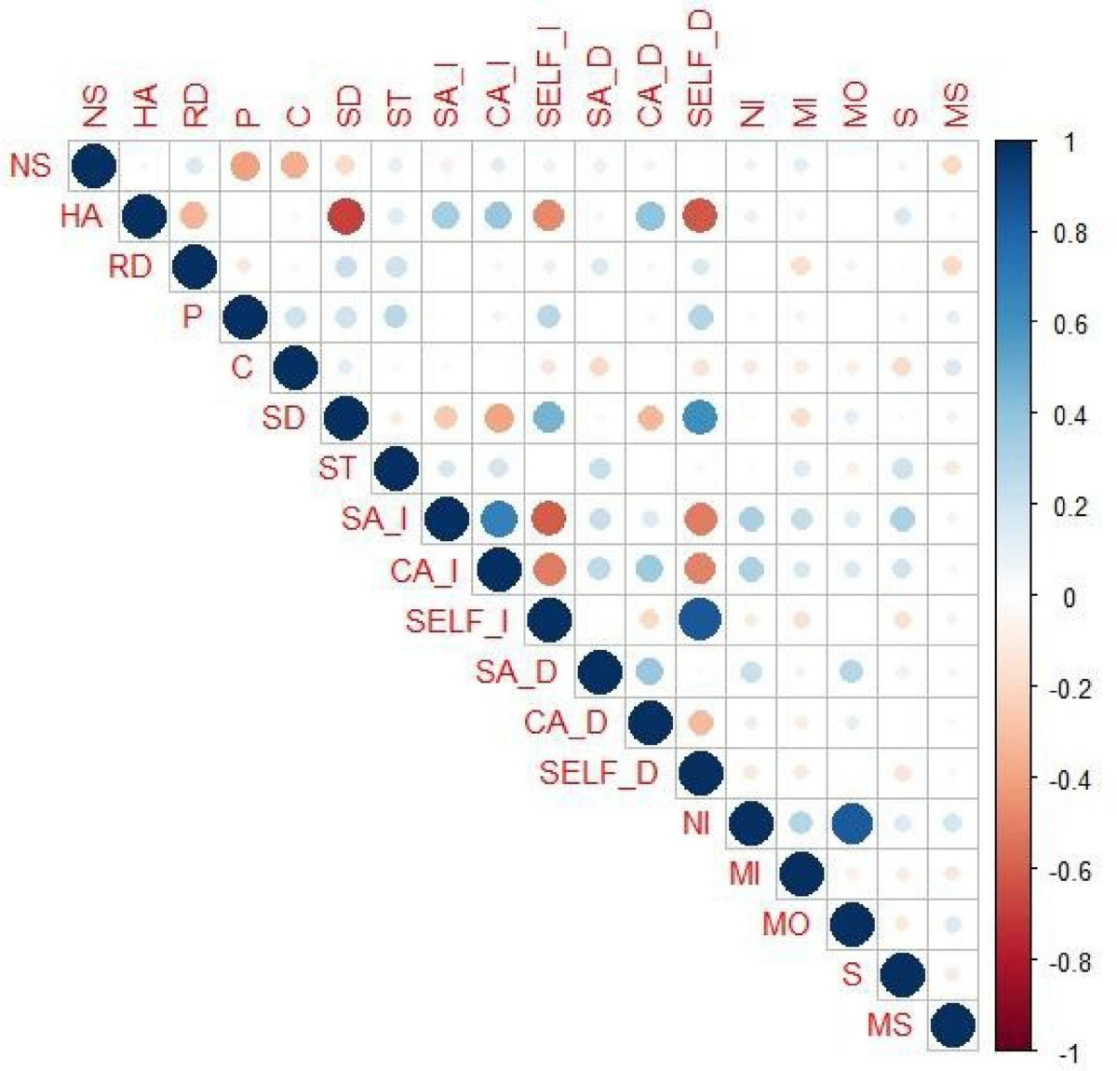
Correlation diagram of personality dimensions, anxiety and injury type. NS, novelty seeking; HA, harm avoidance; RD, reward dependence; P, persistence; C, cooperativeness; SD, self-directedness; ST, self-transcendence; SA-I, somatic anxiety (intensity); CA-I, cognitive anxiety (intensity); SELF-I, self-confidence (intensity); SA-D, somatic anxiety (direction/valence); CA-D, cognitive anxiety (direction/valence); SELF-D, self-confidence (direction/valence); NI, number injury; MI, mild injury; MO, moderate; S, severe; MS, more severe.

Figure 3 presents the correlogram showing the relationships between the severity of sports injuries and personality and anxiety dimensions. The results indicate that minor injuries correlate negatively with Reward Dependence (*r* = −.176; *p* = .048) and Self-Direction (*r* = −.193; *p* = .03). Serious injuries are negatively related to Cooperativeness (*r* = −.181; *p* = .042) and positively related to Self- Transcendence (*r* = .196; *p* = .028) and Harm Avoidance (*r* = .182; *p* = .042). Likewise, athletes who have experienced very serious injuries show a negative association with the temperamental dimension of Novelty Seeking (*r* = −.181; *p* = .043) and Reward Dependence (*r* = −.220; *p* = .013).

On the other hand, the number of injuries is positively associated with the intensity of somatic anxiety (*r* = .331; *p* = .001), cognitive anxiety (*r* = .312; *p* = .001) and somatic anxiety direction (*r* = .234; *p* = .008). In addition, somatic anxiety (intensity) is related to the presence of minor injuries (*r* = .223; *p* = .012) and serious injuries (*r* = .319; *p* = .002); while interpreting somatic anxiety (valence/direction) as a facilitator of athletic performance is associated with moderate injuries (*r* = .275; *p* = .002). Athletes with a tendency to experience cognitive anxiety (intensity) show a positive association with the presence of severe injuries (*r* = .195; *p* = .029).

### 4.3 Predictive analysis

The results of the multinomial logistic regression analysis, presented in Table 2, reveal the predictive contribution of athletes’ personality dimensions to injury severity, using the no-injury group as the reference category. Anxiety variables were not included in the final model, as they did not show a statistically significant contribution to predicting injury severity. The model showed a deviance of 86.400, indicating an acceptable level of lack-of-fit to the observed data. The AIC value was 150.000, reflecting a balance between model fit and parsimony. The McFadden's pseudo-*R*^2^ was 0.508, suggesting that the model accounted for approximately 50.8% of the variability in injury severity among athletes.

**Table 2:** Multinomial regression coefficients for injury severity according to personality.

Injury severity contrast	Predictor	*β*	95% Confidence interval		*p*
			Lower	Upper	
MI-WI	Constant	119.148	119.10	119.19	<.001
	NS	−.444	−.975	.086	.101
	HA	−2.245	−2.909	−1.582	<.001
	RD	−.622	−.961	−.283	<.001
	P	−.053	−.667	.560	.865
	C	−.581	−1.173	.009	.054
	SD	−1.580	−2.314	−.846	<.001
	ST	.671	.098	1.244	.022
MO-WI	Constant	−116.842	−127.674	−106.010	<.001
	NS	−0.390	−.962	.181	.181
	HA	1.571	1.153	1.989	<.001
	RD	0.828	.248	1.407	.005
	P	1.258	.537	1.979	<.001
	C	0.506	−.022	1.034	.060
	SD	1.110	.387	1.833	.003
	ST	−0.636	−1.017	−.255	.001
S-WI	Constant	−115.395	−126.068	104.722	<.001
	NS	−.486	−1.071	.097	.103
	HA	1.600	1.159	2.040	<.001
	RD	.777	.194	1.361	.009
	P	1.146	.420	1.873	.002
	C	.462	−.074	1.000	.091
	SD	1.222	.492	1.953	.001
	ST	−.557	−.940	−.173	.004
MS-WI	Constant	−15.917	−16.019	−15.815	<.001
	NS	−2.944	−4.577	−1.312	<.001
	HA	2.165	.624	3.706	.006
	RD	−.241	−1.073	.590	.569
	P	−.822	−2.074	.430	.198
	C	−.287	−.958	.383	.402
	SD	2.511	.738	4.285	.005
	ST	−1.258	−2.067	.449	.002

NS, novelty seeking; HA, harm avoidance; RD, reward dependence; P, persistence; C, cooperativeness; SD, self-directedness; ST, self-transcendence; WI, without injury; MI, mild injury; MO, moderate; S, severe; MS, more severe.

The results indicated that, compared with uninjured athletes, the likelihood of sustaining a mild injury was negatively associated with Harm Avoidance [*β* = −2.246, 95% CI (−2.909, −1.583), *p* < .001], Reward Dependence [*β* = −0.623, 95% CI (−0.962, −0.284), *p* < .001], and Self-Directedness [*β* = −1.581, 95% CI (−2.315, −0.846), *p* < .001], whereas Self-Transcendence was positively associated with mild injury occurrence [*β* = 0.672, 95% CI (0.099, 1.245), *p* = .022].

For moderate injuries, significant positive associations were observed for Harm Avoidance [*β* = 1.572, 95% CI (1.153, 1.990), *p* < .001], Reward Dependence [*β* = 0.828, 95% CI (0.248, 1.408), *p* = .005], Persistence [*β* = 1.259, 95% CI (0.538, 1.979), *p* < .001], and Self-Directedness [*β* = 1.110, 95% CI (0.387, 1.834), *p* = .003]. In contrast, Self-Transcendence was negatively associated with moderate injury risk [*β* = −0.637, 95% CI (−1.018, −0.256), *p* = .001]. A comparable pattern emerged for severe injuries, with Harm Avoidance [*β* = 1.600, 95% CI (1.160, 2.040), *p* < .001], Reward Dependence [*β* = 0.778, 95% CI (0.194, 1.362), *p* = .009], Persistence [*β* = 1.147, 95% CI (0.421, 1.873), *p* = .002], and Self-Directedness [*β* = 1.223, 95% CI (0.493, 1.953), *p* = .001] being positively associated with injury occurrence, whereas Self-Transcendence again showed a significant negative association [*β* = −0.557, 95% CI (−0.940, −0.174), *p* = .004].

Finally, very severe injuries displayed a distinct predictor profile. Novelty Seeking emerged as a strong negative predictor [*β* = −2.945, 95% CI (−4.577, −1.312), *p* < .001], indicating that lower levels of this trait substantially increase the probability of sustaining very severe injuries. In addition, Harm Avoidance [*β* = 2.166, 95% CI (0.624, 3.707), *p* = .006] and Self-Directedness [*β* = 2.512, 95% CI (0.739, 4.285), *p* = .005] were positively associated with very severe injuries, while Self-Transcendence showed a significant protective effect [*β* = −1.258, 95% CI (−2.068, −0.449), *p* = .002].

## 5 Discussion

The results obtained in this study confirm that psychosocial variables play a significant role in the occurrence and severity of sports injuries, an aspect that had already been proposed in classical theoretical models such as the stress-injury model by Andersen and Williams (20) or Olmedilla-Zafra and García-Mas (22). These models maintain that individual differences, together with levels of anxiety and coping resources, modulate the athlete's response to stressful situations, increasing or reducing the probability of injury. In this vein, the novel proposal of this study lies in the integration of this framework with Cloninger's (32) psychobiological theory of personality, which allows for the joint analysis of temperamental (genetic) and character (social) dimensions in relation to vulnerability to injury. This combination, based on both models, enriches the approach for a better understanding by showing that factors such as emotional regulation, self-direction or harm avoidance, added to somatic and cognitive anxiety, shape a psychological profile that can significantly influence the injury history of elite athletes.

In relation to the first objective of the study—to analyze personality and anxiety dimensions in athletes according to injury history and severity—the descriptive results reveal consistent patterns that confirm the relevance of individual differences in injury processes. In particular, it is observed that novelty seeking is significantly reduced in athletes who have suffered very serious injuries. This decrease can be understood as an adaptive response after injury, insofar as it implies a lower tendency to explore and take risks, consistent with previous research (47). However, it should also be considered that this reduction may reflect a process of motivational inhibition derived from the traumatic experience of the injury, rather than a previous stable trait. In contrast, Persistence appears to be higher in athletes with severe and moderate injuries. Although this dimension has been linked to resilience, sustained motivation and capacity for effort (48), in the context of sports injuries it could take on a less adaptive nuance. Thus, a high level of persistence could reflect a tendency to overexert oneself and return to practice too quickly, which increases the likelihood of relapse or chronicity of the injury. This ambivalence highlights the need to interpret personality dimensions dynamically, drawing on models such as Cloninger's (32); however, the analyses performed, the size of the group with very serious injuries, and the cross-sectional design of the study do not allow us to establish whether these dimensions play a predictive role in the occurrence of injuries.

When integrating the descriptive results on anxiety with the graphical representation, patterns emerge that enrich the understanding of the first objective of the research. Figure 1 shows significant differences in somatic anxiety between injured and uninjured athletes. Unexpectedly, athletes with severe injuries had lower somatic anxiety scores compared to those who had experienced milder injuries. This finding suggests that, after a very serious injury, the physiological symptoms of anxiety may become less relevant than physical pain and rehabilitation processes, as well as contextual factors such as starting or substitute status in competition. In addition to this, Figure 2 and the descriptive data in Table 1 indicate that cognitive anxiety is significantly higher in injured athletes, with a moderate effect size. This reflects that concerns and doubts about performance are a central component of injury in an athlete, in line with the multidimensional model of anxiety by Martens et al. (43). Thus, while somatic anxiety appears to diminish in the context of serious injuries, cognitive anxiety intensifies, probably as an expression of fear of failure, loss of competitive status, and uncertainty about the recovery process (25).

With regard to the second objective of the research, aimed at exploring the association between personality dimensions, anxiety and type of injury, the results show distinctive patterns. Thus, minor injuries show negative correlations with reward dependence and self-direction, suggesting that athletes who are less motivated by external approval and have less self-regulation capacity may be more protected against minor injuries. On the other hand, severe injuries are positively related to Self-Transcendence and Harm Avoidance, as well as negatively related to Cooperativeness, indicating that the experience of severe injuries could activate self-protection strategies and, at the same time, limit collaborative interaction within the sporting context. Harm Avoidance, traditionally associated with cautious behaviour and sensitivity to punishment (29, 30, 32), could translate into greater attention to physical care or preventive behaviours; while Self-Direction, for its part, has been linked to self-regulation and emotional stability (8), which may favour more prudent decisions in situations of physical risk. The negative associations observed between Novelty Seeking and very serious injuries reinforce the idea that exposure to high-risk experiences and the propensity for exploration are reduced after severe injuries, aligning with previous studies that highlight how temperament influences susceptibility to injury (32, 47) and with the results discussed above.

On the other hand, correlations between anxiety and injuries provide a better understanding of the underlying psychological mechanisms. The number of injuries is positively associated with somatic and cognitive anxiety, both in intensity and direction, supporting multidimensional models of sports anxiety (30, 43, 44). These associations suggest that both cognitive concern and physiological arousal are central factors in the experience of injury, increasing vulnerability to further injury or affecting recovery. Furthermore, the negative correlations between self-confidence and anxiety dimensions reflect how perceptions of competence and personal control can act as a buffer against the adverse psychological effects of injury (25, 49). Taken together, these findings reinforce the hypothesis that sports injuries are not only related to physical factors but are also deeply mediated by personality traits and emotional responses, providing a solid basis for psychological interventions aimed at injury prevention and management in elite athletes.

Finally, the third predictive analysis made it possible to identify which personality dimensions carry the greatest explanatory weight in relation to injury occurrence and severity, representing one of the most relevant contributions of the present study. The results of the multinomial logistic regression model indicated that, when all variables were considered simultaneously, anxiety-related variables did not make a statistically significant contribution and were therefore excluded from the final model. In contrast, several personality dimensions, encompassing both temperamental and character-related traits, emerged as significant predictors of injury severity.

Specifically, temperamental dimensions associated with emotional and behavioral regulation, such as Harm Avoidance and Reward Dependence, together with character dimensions related to self-regulation and identity, namely Self-Directedness and Self-Transcendence, showed differential effects depending on injury severity. These findings extend previous empirical evidence, as most prior research has relied primarily on bivariate correlational analyses without examining the joint explanatory power of multiple personality dimensions on injury severity (7).

From an applied perspective, identifying these predictors is particularly relevant, as it allows a shift from purely descriptive approaches toward prevention-oriented models based on psychological risk profiles. The results suggest that athletes with lower levels of Harm Avoidance, Reward Dependence, and Self-Directedness are less likely to sustain mild injuries, possibly due to more adaptive self-regulation and more effective coping strategies in risky situations. Conversely, higher levels of Self-Transcendence were associated with an increased likelihood of mild injuries, which may reflect greater personal involvement and a tendency to exceed physical limits.

Regarding moderate and severe injuries, the combination of higher Harm Avoidance, Persistence, Reward Dependence, and Self-Directedness appears to characterize athletes who are highly committed and perseverant, but who may remain exposed to physical risk under conditions of sustained competitive demands. Finally, very severe injuries showed a distinct predictor profile, marked by low Novelty Seeking and elevated Harm Avoidance and Self-Directedness, suggesting that a more rigid, conservative, and self-demanding personality style may increase vulnerability to particularly severe physical consequences (29, 31). Overall, these findings underscore the importance of conceptualizing personality as a dynamic and multifactorial system in the understanding and prevention of sports injuries.

However, it is important to recognize the limitations of this research. The sample size, although relevant, limits the generalizability of the results, especially when analysing the severity and type of injuries, where some categories were represented by a small number of cases. Furthermore, the cross-sectional design prevents the establishment of causal relationships, so it would be advisable to implement longitudinal designs that allow for the examination of how variations in personality and anxiety influence the incidence and evolution of injuries over time. The implementation of prospective longitudinal designs should be prioritized. Following cohorts of elite athletes across one or more competitive seasons, assessing personality traits and baseline anxiety levels prior to competition, and systematically recording injury incidence over time would enable the establishment of clear temporal sequences and strengthen conclusions regarding causality. Such designs would substantially enhance the predictive validity of psychosocial injury-risk models. Another aspect to consider is the use of self-report questionnaires, which, although validated instruments, may be subject to social desirability or memory biases. It is recommended that future analyses differentiate between injury subtypes, such as traumatic vs. overuse injuries or muscular vs. joint injuries. This distinction would help clarify whether certain personality traits (e.g., Novelty Seeking or impulsivity) are more strongly associated with acute, risk-related injuries, whereas other traits (e.g., high Harm Avoidance, chronic muscle tension, or poor effort self-regulation) may be more relevant for overload-related injuries. Understanding these mechanisms would significantly refine theoretical models linking personality to injury vulnerability. Future research could complement them with objective measures, more comprehensive medical records, or qualitative approaches that delve deeper into the subjective experience of injury.

In short, the results underscore that sports injuries should be understood as a complex phenomenon involving physical, psychological, and social factors. Recognizing the relevance of personality and anxiety dimensions not only broadens our understanding of the problem but also opens up new avenues for intervention that can contribute to the overall well-being and sustainable performance of elite athletes. From an applied perspective, these results have direct implications for the design of injury prevention programmes in elite sport. On the one hand, systematic psychological assessment should be incorporated into medical and physical training protocols, allowing risk profiles to be identified based on personality and anxiety.

Sports psychologists could work on reducing somatic anxiety through activation control techniques (e.g., diaphragmatic breathing, progressive relaxation) and on managing cognitive anxiety through cognitive restructuring and coping skills training (24). In addition, promoting self-confidence through realistic goal setting, constructive feedback, and visualization can be key to reducing the incidence of injury and facilitating more effective recovery. In terms of personality, understanding that high persistence can have a dual perspective is essential for coaches and medical staff. While this trait promotes adherence to rehabilitation programmes, it can also lead to premature returns if not properly regulated. A psychoeducational intervention aimed at modulating this tendency can balance determination with prudence. Similarly, promoting self-direction and cooperativeness can help athletes take active responsibility for their recovery process and work more collaboratively with the multidisciplinary team.

## Data Availability

The raw data supporting the conclusions of this article will be made available by the authors, without undue reservation.
